# Evaluation of eef1a1l1 expression stability and promoter-driven reporter activity during zebrafish development

**DOI:** 10.17912/micropub.biology.002410

**Published:** 2026-09-14

**Authors:** Nicole A. Aponte-Santiago, Nicole Suren, Yingxin Su, Coral N. Halanych, Maile N. Matubrew, Erin Song, Ian A. Swinburne, David Q. Matus, Daniel E. Wagner

**Affiliations:** 1 Department of Obstetrics, Gynecology and Reproductive Science, Center for Reproductive Sciences, UC San Francisco, San Francisco, CA, United States; 2 Eli and Edythe Broad Center for Regeneration Medicine and Stem Cell Research, UC San Francisco, San Francisco, CA, United States; 3 Department of Molecular and Cell Biology, UC Berkeley, Berkeley, CA, United States; 4 Janelia Research Campus, Ashburn, VA, United States

## Abstract

Housekeeping genes are widely used as experimental references for gene expression, yet many exhibit substantial variation within and between cell types. Using Zebrafish Meta Atlas Project (ZMAP), we found *eef1a1l1* to be ubiquitously and highly expressed across zebrafish embryonic cell types, developmental stages, and studies. Transgenic reporters driven by 2.2-kb or 2.6-kb *eef1a1l1* -derived regulatory sequences showed broad embryonic expression, and single-cell profiling revealed that reporter abundance covaried with that of the endogenous *eef1a1l1* transcript. These findings further support *eef1a1l1* as a reference for embryonic gene expression and the use of its regulatory sequence as a practical, multipurpose transgenic driver.

**Figure 1. Evaluation of eef1a1l1 as a broadly expressed housekeeping gene:** (A) Left, scatterplot of normalized dispersion versus log-transformed mean TPM (transcripts per million) expression counts for all genes in ZMAP. Genes are colored by classification criteria: *stable* (normalized dispersion values < 0, blue), *abundant* (top 0.1% of mean expression, red), and *stable + abundant* (satisfying both criteria, magenta). Labels for example housekeeping genes are shown. Right, top candidate housekeeping genes, jointly ranked by dispersion and abundance (see also Extended Data Table 1). (B) UMAP overlay of *eef1a1l1* transcript counts expressed as log-normalized TPM counts, showing high and broadly distributed expression across cells. (C) Dot plot of *eef1a1l1* expression across CellType groups, stratified by developmental time window (left) and contributing study (right). Dot color and size reflect average expression and fraction of expressing cells, respectively. Omitted groups with insufficient cell numbers (<10 cells or <1% of the group’s total representation in the integrated dataset) are depicted as gray circles. (D) Construct design (not to scale). Transcription start site (TSS), ATG start codon, and genomic positions (GRCz11) are indicated. (E) Representative confocal maximum-intensity projection of a live Tg( *2.2eef1a1l1:h2b-mCherry2* ) embryo at 24 hours post-fertilization (hpf). Nuclear mCherry2 signal is observed across diverse tissues. Scale bar, 200 µm. Labels indicate major anatomical structures: fb (forebrain), mb (midbrain), mhb (midbrain-hindbrain boundary), hb (hindbrain), evl (enveloping layer), ot (otic vesicle), ret (retina), len (lens), hg (hatching gland), ysl (yolk syncytial layer), som (somites), tb (tailbud), and sc (spinal cord). (F) Projection of an inDrops scRNA-seq dataset generated from Tg( *2.2eef1a1l1:h2b-mCherry2* ) embryos onto the ZMAP reference using the zmap-tools annotation pipeline. A total of 8,587 cells were annotated. Assigned labels (n=46) are indicated and colored according to germ layer groupings. (G) Pseudobulk mean raw counts per annotated cell type (n=46) for mCherry2 and *eef1a1l1* . Point size scales with log10 cell number per group; colors as in (F). Dashed line, linear regression fit. Inset: Spearman's rho (ρ), P-value, log-log slope and R ^2^ . Table: Spearman’s rho against *eef1a1l1* for mCherry2 and indicated control genes. (H) Representative spinning disk confocal maximum-intensity projections of live Tg(p14a.2.2 *eef1a1l1* :mSG-sec61b) (left) and Tg(p14a.2.6 *eef1a1l1* :mSG-sec61b) (right) embryos at 72 hpf of the trunk/flank musculature just posterior to the yolk extension. Scale bar, 100 µm. (I) Quantification of n > 17 projections per allele, n.s. not significant (p = 0.26, Mann–Whitney test).

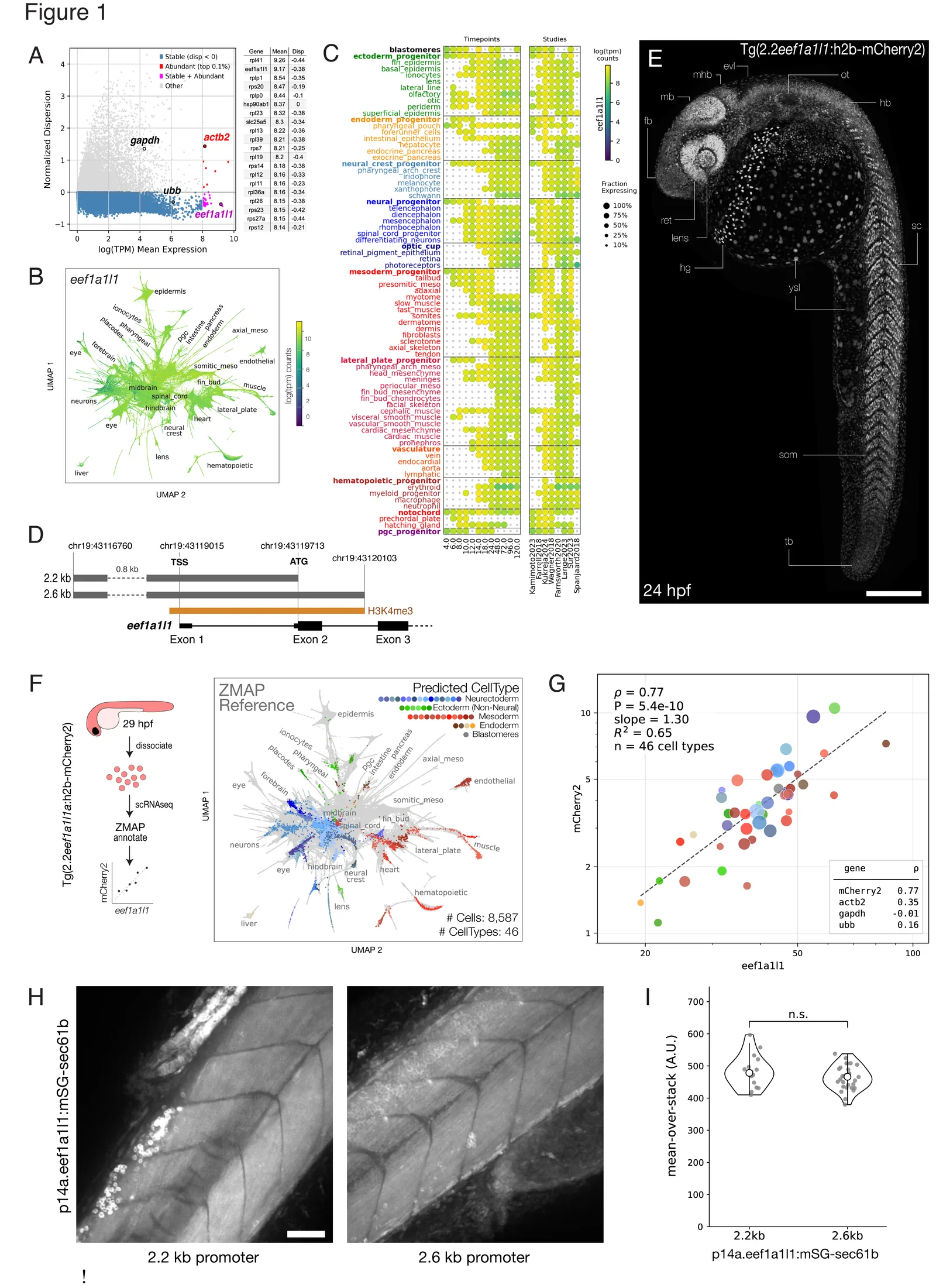



## Description

Housekeeping genes are widely used as reference controls for gene expression analyses and as transgenic drivers. Across model systems, a relatively small number of such genes have become established for this purpose, owing to broad expression and relative stability across life stages and biological contexts. In zebrafish, commonly used references include *ubiquitin B* ( *ubb* ), *beta-actin* ( *actb1* ; *actb2* ), and *glyceraldehyde-3-phosphate dehydrogenase* ( *gapdh* ) (Burket et al. 2008; Mosimann et al. 2011; Choe et al. 2021). *Translation elongation factor 1-alpha 1-like 1* ( *eef1a1l1* ) has been repeatedly evaluated against other reference genes and ranks among the most stable across developmental stages and tissues (Tang et al. 2007); sex, tissues, stages, and perturbations (McCurley and Callard 2008; Xu et al. 2016); and across organs in transgenic and wild-type backgrounds (Rassier et al. 2020). Absolute transcript numbers in early embryonic stages have also been reported (Ligunas and Materna 2026). Much of this supporting evidence, however, derives from bulk profiling measurements that cannot resolve whether expression stability is preserved within or between cell types. Whole-embryo single-cell RNA sequencing (scRNA-seq) provides an opportunity to evaluate housekeeping genes using empirical measures of expression abundance and stability across individual cells. Here, we interrogated ZMAP (Aponte-Santiago et al. 2026), a zebrafish scRNA-seq meta-atlas comprising 798,790 cells from eight published datasets (Farrell et al. 2018; Spanjaard et al. 2018; Wagner et al. 2018; Farnsworth et al. 2020; Kamimoto et al. 2023; Sur et al. 2023; Kukreja et al. 2024; Lange et al. 2024) to systematically evaluate gene expression abundance and stability across cell types, developmental stages, and technologies.

To assess housekeeping-like characteristics in an unbiased manner, we compared normalized dispersion and global mean expression across all 36,365 genes in ZMAP (Extended Data Table 1). A total of 30 genes satisfied criteria for both stability (normalized dispersion < 0) and high mean expression across cells (top 0.1%) ( Fig. 1A ). Among these 30 genes, the majority (27/30) encoded ribosomal proteins. *eef1a1l1* ranked as the top non-ribosomal gene meeting both criteria, while other references *ubb* , *actb2* , and *gapdh* satisfied at most one of these criteria. We next confirmed broad, stable *eef1a1l1* expression across the ZMAP developmental manifold ( Fig. 1B ). Across ZMAP cell types stratified by developmental stage and originating study, *eef1a1l1* showed high mean expression and was detected in a large fraction of cells ( Fig. 1C ).

EF1a regulatory elements are widely used as constitutive transgenic drivers across model systems, yet zebrafish *eef1a1l1* -based reagents remain less extensively developed. Some studies have utilized a *Xenopus* Ef1a-derived 500-bp driver that was later found to be prone to silencing (Johnson and Krieg 1994; Kawakami et al. 2004; Thummel et al. 2006; Burket et al. 2008). A subsequent line based on 1.4 kb of zebrafish *eef1a1l1* upstream sequence drove broad expression, but has not been widely adopted (Moon et al. 2013). Motivated by this gap, we set out to develop additional transgenic reagents based on this locus. We evaluated two candidate transgenic drivers: (1) a 2.2-kb genomic sequence upstream of the endogenous *eef1a1l1* start codon, encompassing the promoter, 5′ UTR, and first intron; and (2) a 2.6-kb sequence comprising the same upstream regions and extending into the second intron to encompass a broad H3K4me3 peak ( Fig. 1D ) (Baranasic et al. 2022).

We first tested the ability of the 2.2-kb construct to drive expression in live embryos using Tol2 transgenics. Live confocal imaging of Tg( *2.2eef1a1l1:h2b-mCherry2* ) embryos at 24 hours post-fertilization (hpf) revealed reporter signals that spanned multiple tissues and anatomical regions ( Fig. 1E ), demonstrating broad expression activity. To evaluate the 2.2-kb driver at single-cell resolution, we used zmap-tools to annotate an external inDrops scRNA-seq dataset collected from Tg( *2.2eef1a1l1:h2b-mCherry2* ) embryos at 29 hpf (see Methods). Projection onto the ZMAP reference enabled annotation of 8,587 transgenic cells across 46 cell types, each mapping to discrete regions of the UMAP embedding ( Fig. 1F ). Because raw housekeeping gene counts vary with RNA content, cell size, and transcript capture, we asked whether raw pseudobulked mCherry2 counts scaled with those of endogenous *eef1a1l1* across cell types. This analysis revealed a strong positive relationship that was approximately linear in log-transformed counts (Spearman’s ρ=0.77, P=5.4e-10; slope=1.3, R ^2^ =0.65) and was stronger than correlations between mCherry2 and *actb2* , *gapdh* , or *ubb* ( Fig. 1G ). These data demonstrate that reporter transcript abundance driven by the 2.2-kb sequence broadly covaries with that of endogenous *eef1a1l1* across diverse embryonic cell types.

We next compared the performance of the 2.2-kb and 2.6-kb drivers using the standardized safe-harbor locus: phiC31 Integrase Genomic Loci Engineered for Transgenesis 14a (“pIGLET14a”) (Lalonde et al. 2024), and an ER-localized monomeric StayGold fluorescent reporter (Diez et al. 2022; Tschanz et al. 2025). Live imaging of heterozygous Tg(p14a.2.2 *eef1a1l1* :mSG-sec61b) or Tg(p14a.2.6 *eef1a1l1* :mSG-sec61b) carriers at 72 hpf revealed broad reporter expression across tissues in both lines ( Fig. 1H ), with no significant difference in mean fluorescence intensity across confocal z-stacks ( Fig. 1I ). Thus, at the pIGLET14a locus, both drivers displayed similar performance with respect to overall expression level and tissue distribution.

While both the 2.2-kb and 2.6-kb sequences appear capable of driving broad transgene expression, we recommend the 2.6-kb sequence for future work as it retains a larger portion of the endogenous promoter-associated region. For Tol2-based transgenics, we recommend the evaluation of multiple independent founders to account for integration and founder-specific effects; alternatively, this construct performs well with pIGLET-targeted integration. To support community usage, plasmids generated in this study are available through Addgene, and stable transgenic lines are available by direct request. Together, these findings support the use of *eef1a1l1* as a stable endogenous reference for zebrafish embryonic gene expression and further demonstrate the use of its upstream regulatory sequence as a practical and broad transgene driver.

## Methods

**Analysis of scRNA-seq Data**

Processed scRNA-seq data were obtained from the Zebrafish Meta Atlas Project (ZMAP) (Aponte-Santiago et al. 2026), which comprises PRJNA417290, PRJNA929041, PRJNA445487, PRJNA1123686, PRJNA564810, PRJNA940501, PRJNA321866, and PRJNA606682. Additional public inDrops scRNA-seq data were obtained from GSE326623 (PRJNA1446332). Analyses used zmap-tools [v0.2.2.2], scanpy [v1.12.1], scipy [v1.16.3], numpy [v2.0.2], and matplotlib [v3.10.0] in Python [v3.12.13]. Housekeeping gene statistics (normalized dispersion and normalized transcript abundance) were calculated using scanpy.pp.highly_variable_genes and plotted using matplotlib ( Fig. 1A ). The UMAP embedding and dotplot ( Fig. 1B- C) were generated using scanpy.pl.embedding and zmap.dotplot.gene_view, using all cells in the ZMAP reference. zmap-tools was also used to pre-process, project, and annotate scRNA-seq data collected from Tg( *2.2eef1a1l1:h2b-mCherry2* ) embryos at 29 hpf using zmap.predict.annotate_with_zmap ( Fig. 1F ); annotation was performed at the “CellType” level of the ZMAP ontology and p_thresh was set to 0.5. For pseudobulk correlation analyses ( Fig. 1G ), cells were grouped by their predicted “CellType” and mean raw UMI counts per cell for each gene were calculated. Concordance between mCherry2 and *eef1a1l1* across cell types was quantified by Spearman's rank correlation (scipy), and by linear regression (numpy) of log10 counts for both genes.

**Generation of Tg( *2.2eef1a1l1:h2b-mCherry2* )**

Using primers indicated below, a 2.2-kb genomic fragment upstream of the predicted *eef1a1l1* translation start site was amplified from purified zebrafish AB strain genomic DNA using Phusion polymerase. PCR cycling conditions: 98°C for 1 min; 35 cycles of 98°C for 10 s and 72°C for 3 min; followed by 72°C for 5 min. The resulting amplicon was ligated into a pMTB vector backbone containing superfolder GFP (Addgene #112225) to replace the pMTB *actb2* promoter with that of *eef1a1l1,* yielding Tg(2.2 *eef1a1l1:sfGFP* ). The *eef1a1l1* promoter was subsequently subcloned to generate Tg( *2.2eef1a1l1:h2b-mCherry2* ) via a 3-fragment NEB HiFi DNA assembly. The cloned 2.2-kb regulatory fragment contained an approximately 800-bp deletion within the upstream region relative to the corresponding AB and TU reference genome sequences. The complete experimentally tested sequence is available via Addgene #254876. Tg( *2.2eef1a1l1:h2b-mCherry2* ) was used for transgenesis by co-microinjection of single-cell stage zebrafish embryos with Tol2 mRNA (50 ng/µL) (Kawakami and Shima 1999) and plasmid DNA (10 ng/µL). Transgenic founders were identified by live fluorescence imaging. Primer sequences used for cloning the 2.2-kb fragment upstream of *eef1a1l1* (NM_131263) are F: GGCCAAAGGTTTGACAACAT, R: GATTGATAAGTTTCTGCGGAC.

**Generation of pIGLET Transgenics**

To extend the *eef1a1l1* promoter from 2.2 to 2.6 kb, additional sequence predicted from the ZFIN GRCz11 annotation (chr19 43119713-43120103) was synthesized (Twist Biosciences) and subcloned into the 2.2-kb driver plasmid. Because this region included the first exon, a potential translation initiation codon (ATG) was changed to AGG to prevent expression of a truncated *eef1a1l1* product. To generate plasmids MGCO-04 (2.2-kb version) and MGCO-27 (2.6-kb version) for mStayGold:sec61b constructs, we synthesized an iCodon-optimized (Diez et al. 2022) (https://bazzinilab.shinyapps.io/icodon/) version of sec61b based on the Sec61 amino acid sequence from the Davidson collection plasmid pmApple-Sec61-C-18 (Tsuda et al. 2023) with overhangs for NEBuilder HiFi DNA assembly cloning (NEB). First, we added the mStayGold c4 linker (Hirano et al. 2022) to a base plasmid (pDQM102) with a 2.2-kb *eef1a1l1:mStayGold* insert using a KLD mutagenesis reaction with primers, C4-KLD-F AGCGCGAGTGCTGTAGGAGCCTCCGGAGCCTCCG, and C4-KLD-R AGGTTCATGCCAGGGCAAGTGAGCCTCCAGGGTTTCG. Next, to generate plasmid MGCO-04 we performed an NEBuilder HiFi DNA assembly reaction with PCR-amplified backbone using primers, 3p-linker-DQM-SV40-F taaatcgatgatgatccagacatgataag and 3p-linker-DQM-R TGGCGCGCCGGAGGCTC and the synthesized sec61b block. To add the additional *eef1a1l1* promoter sequence to generate MGCO-27, we amplified the backbone of MGCO-04 using primers pDQM-ef1a-extraF tttgttaatcAGgccgccacCATGGGAGCATCGGG and pDQM-ef1a-extra-R ccgtaatgactaggccctcgag and primers pDQM133-ef1a-exonF cggctcgagggcctagtcattac and pDQM133-ef1a-exon-R CCATGgtggcggcCTgattaa. Plasmid sequence was verified by whole plasmid sequencing. Plasmids MGCO-04 and MGCO-27 were used for transgenesis by co-microinjection of single-cell stage zebrafish embryos with PhiC31 mRNA (25 ng/ul) and plasmid DNA (25 ng/ul). Transgenic founders were identified by PCR screening of sperm followed up by live fluorescence imaging. Genotyping to confirm integration was performed using the following primers: DM323, CCTAGCGCGAGTGCTGT and DM320, CTGACTGCGTCACTTTGACAC for insertion at the pIGLET14a locus and DM235 acaccgtcttactctaaagaaacacg and DM387 CCATATCTCCAGTCAGAACAG to confirm the amplicon difference between the 2.2-kb and 2.6-kb promoter.

**Confocal Microscopy and Image Quantification**

Imaging data for the Tol2-based Tg(2.2 *eef1a1l1* :h2b-mCherry2) ( Fig. 1E ) were collected on a Leica Stellaris 5 scanning confocal microscope. Shown is a tiled maximum intensity projection collected using an HC-PL-APO-L 10x/0.40 CS2 objective (Leica). Imaging data for the phiC31-based pIGLET alleles Tg(p14a.2.2 *eef1a1l1* :mSG-sec61b) and Tg(p14a.2.6 *eef1a1l1* :mSG-sec61b) ( Fig. 1H ) were collected from heterozygous outcrossed *casper* embryos reared at 28˚C to 72hpf. Larvae were anesthetized in tricaine and mounted in a glass bottom 96-well dish (Ibidi 89626). Imaging was performed with a Yokogawa X Spinning Disk confocal microscope on a Nikon TiE frame using a Hamamatsu EM-CCD camera. Single channel z-stacks of the trunk/flank musculature just posterior to the yolk extension were acquired with the 488nm laser (100% power, 150 ms). Z-stacks comprised a 60 µm volume sampled every 5 µm, or 13 planes per z-stack. Acquisition settings were identical for all data collection. A single field of view was captured per animal. Background reference acquisitions were collected using identical settings, to capture camera offset and read noise (laser off) and laser-induced background on an empty field of view (laser on). Representative maximum intensity projections are shown in Figure 1H .

**Image Quantification and Statistical Analyses**

Image analysis was performed in Python 3.12.2 (numpy 2.4.2, scipy 1.17.1, pandas 3.0.0, scikit-image 0.26.0, matplotlib 3.10.9, nd2 0.11.3) using scripts co-authored with Claude Opus 4.8 (Anthropic). A per-pixel total background (mean of the laser-on reference frames) was subtracted and the result clipped at zero for each z-stack. For each z-stack we quantified the mean-over-stack: the mean of all voxels of the background-subtracted stack. This metric is linear (proportional to total signal divided by the number of z-planes), independent of projection choice, and insensitive to the number of optical sections, avoiding the non-linearity and z-plane-count sensitivity of max-intensity-projection means. Per-fish mean-over-stack values were compared between the 2.2 kb (n = 17) and 2.6 kb (n = 30) promoter constructs using a two-sided Mann–Whitney U test (primary), with a Welch *t*-test and Cohen's *d* effect size reported alongside. The two promoter lengths were statistically indistinguishable (482 ± 49 vs 464 ± 37 A.U., mean ± s.d.; Mann–Whitney p = 0.26; Welch *t*-test p = 0.15; Cohen's *d* = −0.43; fold change 0.96X). All analysis code, per-fish measurement tables, and figures are available at https://github.com/dqmatus/piglet14a-sec61b-staygold. The reported statistics and quantification plot can be regenerated from the committed measurement tables with `scripts/reproduce_stats.py` (no raw data required). Raw confocal z-stacks are archived on Zenodo (https://doi.org/10.5281/zenodo.21520506) and support full reproduction including the representative image panels.

## Reagents

Zebrafish lines and corresponding ZFIN allele designations and Addgene plasmid accessions described in this study are as follows: casper: *mitfa* ^w2/w2; *mpv17* ^a9/a9; Tg(2.2 *eef1a1l1* :h2b-mCherry2)s5002Tg (Addgene #254876); Tg(2.2 *eef1a1l1* :h2b-mNeonGreen)s5001Tg (Addgene #254877); Tg(p14a.2.2 *eef1a1l1* :mSG-sec61b)bbc459Tg; and Tg(p14a.2.6 *eef1a1l1* :mSG-sec61b)bbc460Tg (Addgene #258401). Transgenic zebrafish lines s5001Tg, s5002Tg, bbc459Tg, and bbc460Tg are available upon request.

## Data Availability

Description: Extended Data Table 1. Top 200 candidate housekeeping genes Genes were identified from ZMAP by filtering for normalized dispersion < 0 and ranked by mean log transformed TPM expression. Columns include expression rank, gene symbol, mean log-TPM, normalized dispersion, Ensembl gene ID, RefSeq mRNA accession, functional category (manually assigned), and full gene name. Data for the top 200 ranked candidate genes are reported. The top 30 genes (ranks 1–30) satisfied both the stability criterion and high expression abundance (top 0.1% of all transcripts). Resource Type: Text. DOI: https://doi.org/10.22002/dpprq-cjx91
